# Implementation of an antimicrobial stewardship program for urinary tract infections in long-term care facilities: a cluster-controlled intervention study

**DOI:** 10.1186/s13756-024-01397-2

**Published:** 2024-04-16

**Authors:** Elisabeth König, Lisa Kriegl, Christian Pux, Michael Uhlmann, Walter Schippinger, Alexander Avian, Robert Krause, Ines Zollner-Schwetz

**Affiliations:** 1 Division of Infectious Diseases, Department of Internal Medicine, Medical University of Graz, Auenbruggerplatz 15, A-8036 Graz, Austria; 2 Geriatric Health Centers of the City of Graz, Graz, Austria; 3 Institute of Medical Informatics, Statistics and Documentation, Medical University of Graz, Graz, Austria

**Keywords:** Nursing home, Healthcare associated infection, Antibiotic stewardship, Urinary tract infections

## Abstract

**Background:**

Widespread inappropriate use of antimicrobial substances drives resistance development worldwide. In long-term care facilities (LTCF), antibiotics are among the most frequently prescribed medications. More than one third of antimicrobial agents prescribed in LTCFs are for urinary tract infections (UTI). We aimed to increase the number of appropriate antimicrobial treatments for UTIs in LTCFs using a multi-faceted antimicrobial stewardship intervention.

**Methods:**

We performed a non-randomized cluster-controlled intervention study. Four LTCFs of the Geriatric Health Centers Graz were the intervention group, four LTCFs served as control group. The main components of the intervention were: voluntary continuing medical education for primary care physicians, distribution of a written guideline, implementation of the project homepage to distribute guidelines and videos and onsite training for nursing staff. Local nursing staff recorded data on UTI episodes in an online case report platform. Two blinded reviewers assessed whether treatments were adequate.

**Results:**

326 UTI episodes were recorded, 161 in the intervention group and 165 in the control group. During the intervention period, risk ratio for inadequate indication for treatment was 0.41 (95% CI 0.19–0.90), *p* = 0.025. In theintervention group, the proportion of adequate antibiotic choices increased from 42.1% in the pre-intervention period, to 45.9% during the intervention and to 51% in the post-intervention period (absolute increase of 8.9%). In the control group, the proportion was 36.4%, 33.3% and 33.3%, respectively. The numerical difference between intervention group and control group in the post-intervention period was 17.7% (difference did not reach statistical significance). There were no significant differences between the control group and intervention group in the safety outcomes (proportion of clinical failure, number of hospital admissions due to UTI and adverse events due to antimicrobial treatment).

**Conclusions:**

An antimicrobial stewardship program consisting of practice guidelines, local and web-based education for nursing staff and general practitioners resulted in a significant increase in adequate treatments (in terms of decision to treat the UTI) during the intervention period. However, this difference was not maintained in the post-intervention phase. Continued efforts to improve the quality of prescriptions further are necessary.

**Trial registration:**

The trial was registered at ClinicalTrials.gov NCT04798365.

**Supplementary Information:**

The online version contains supplementary material available at 10.1186/s13756-024-01397-2.

## Introduction

Antimicrobial resistance is a major threat to human health. The widespread inappropriate use of antimicrobial substances drives resistance development at the individual and population level [1, 2]. Infections due to resistant pathogens are responsible for a high healthcare burden and are estimated to cause over 700.000 deaths annually worldwide– a rate that is projected to rise to 10 million by 2050 [3].

Approximately 2–5% of the population of high-income countries resides in some type of long-term care facilities (LTCFs) [4]. These residents are at increased risk for nosocomial and healthcare-associated infections mostly due to age-related factors such as immunosenescene, decline in functional status, chronic comorbidities and the use of invasive medical devices [5, 6]. Infections at LTCFs are a common cause for residents’ mortality and morbidity associated with a significant socio-economic burden [7].

Antibiotics are one of the most frequently prescribed medications in LTCFs [8, 9]. In a multinational European point-prevalence study, 4.9% of residents received at least one antimicrobial substance on the study day [10]. Over 30% of antibiotics prescribed in LTCFs are for urinary tract infections (UTIs) [11]. Studies have shown that 30–43% of antibiotic courses prescribed in LTCFs were unnecessary [12–14]. In addition, up to 72% of patients with UTIs in LTCFs were shown to be treated with inappropriate antimicrobial drugs based on society guidelines [13]. Antimicrobial stewardship programs targeting prescriptions for residents of LTCFs can therefore be a valuable contribution in the strive to curb antimicrobial resistance [15].

The aim of our study was to increase the number of appropriate antimicrobial treatment courses prescribed for UTIs in LTCFs using a multi-faceted antimicrobial stewardship intervention.

## Materials and methods

### Design

Non-randomized cluster-controlled intervention study.

### Setting

The Geriatric Health Centers Graz are a local institution comprising among others four LTCFs (total of 400 beds). General practitioners who are located off-site are in charge of medical treatments (approximately ten general practitioners per LTCF). These LTCFs were the intervention group. Four LTCFs located in the surroundings of Graz served as control group. Randomisation of LTCFs was not feasible because several general practitioners care for patients in more than one LTCF in Graz. We therefore chose LTCFs in another region as control group to avoid spillover of the intervention. The study was conducted from January 2021 to June 2022.

### Interventions

A multifaceted educational intervention targeting nursing staff as well as physicians was initiated (“Urinary tract infection program”) targeting the following key-points:

1. obtain urine specimen in symptomatic residents when criteria for urine culture have been met (Supplement Table 1).
2. obtain urine specimen using proper techniques to avoid contamination.
3. change indwelling urinary catheters before obtaining urine specimen.
4. prescribe antibiotics only when clinical criteria have been met (Supplement Table 2).
5. prescribe antibiotics according to guideline (Supplement 3).

**Table 1:** Characteristics of long-term care facilities in the control and intervention group

	Control				Intervention				
	LTCF 1	LTCF 2	LTCF 3	LTCF 4	LTCF 5	LTCF 6	LTCF 7	LTCF 8	
Number of beds	124	93	37	59	100	97	104	105	
Ownership	Municipal	Private	Non-profit	Non-profit	Municipal	Municipal	Municipal	Municipal	

**Table 2:** Characteristics of residents with UTIs in long-term care facilities in the control and intervention group

	Control	Intervention	p-value
Age (median, range; years)	85 (49–99)	87 (38–102)	0.064
Female residents (%)	71.7	78.9	0.243
Weight (median, range; kg)	66 (38–135)	59 (38–143)	**0.014**
History of allergy to antiinfectives (%)	5.1	9.5	0.234
Renal impairment (%)	22.2	22.1	0.984
Urologic disease (%)	21.2	22.1	0.880

Bold writing indicates statistically significant results

Main components of the intervention:

- two online sessions of voluntary continuing medical education on the UTI program for treating general practitioners.
- two online meetings with team leaders of nursing staff of all LTCFs discussing the UTI program.
- interactive educational session with all nursing staff onsite at each of the LTCFs led by the principal investigator during the intervention period including information on the aims of the project, on how to recognize UTIs in the elderly and indications for urinary cultures according to the UTI guideline (see supplement).
- onsite trainings on infection control for all nursing staff led by ICP team including information on UTI prevention strategies, on how to recognize UTIs in the elderly and correct techniques for collection of urine specimens.
- educational materials: handouts during educational sessions, written guideline on antibiotic prescribing (supplement 3), videos about different aspects of the UTI program available on the project homepage.
- project homepage used as platform to distribute the guideline (supplement 3) and educational videos.

### Data collection

Baseline data on participating LTCFs were obtained: number of beds and ownership (municipal, private). For data on prescriptions, the nurse responsible was requested to fill in an online case report form (CRF) for all patients with infectious symptoms suggestive of UTI requiring a physician’s opinion. Recorded information included age, sex, indwelling urinary catheter, signs and symptoms suggestive of UTI, duration of symptoms, performance of urinary culture, type of antibiotic treatment, treatment length and dosing, referral to hospital, history of hypersensitivity to antimicrobial substances, pre-existing diagnosis of renal impairment, pre-existing urologic diagnosis, method of communication with the treating physician. The study was divided into a pre-intervention, an intervention and a post-intervention phase (for specific dates see supplement Table 3).

**Table 3:** Summary of results (risk ratios)

	Before intervention RR (95%CI)	During intervention RR (95%CI)	After intervention RR (95%CI)
Inadequate choice of antimicrobial	0.91 (0.63–1.32) *p* = 0.621	0.82 (0.62–1.09) *p* = 0.178	0.76 (0.50–1.14) *p* = 0.182
Inadequate decision to treat	1.13 (0.48–2.67) *p* = 0.784	**0.41 (0.19–0.90)** *p* = 0.025	1.04 (0.27–4.10) *p* = 0.951
Quinolone use for UTI without catheter	**0.17 (0.04–0.72)** *p* = 0.017	0.67 (0.22–2.07) *p* = 0.484	**0.18 (0.04–0.89)** *p* = 0.035
Urinary culture performed	6.18 (0.80–47.92) *p* = 0.081	**6.89 (1.59–29.83)** *p* = 0.010	n.d.§
Clinical failure	1.77 (0.56–5.55) *p* = 0.329	1.09 (0.45–2.61) *p* = 0.855	n.d.#
Hospital admission due to UTI	0.86 (0.19–3.88) *p* = 0.847	1.21 (0.32–4.61) *p* = 0.777	6.35 (0.82–49.37) *p* = 0.077

n.d. = not done, RR = risk ration

§ In the CG, no urinary cultures were performed in the post-intervention period

# In the CG, no clinical failure was reported in the post-intervention period

Bold writing indicates statistically significant results

To assess the appropriateness of prescriptions in terms of antimicrobial choice and in terms of decision to treat, two independent blinded infectious disease specialists (LK, EK) reviewed each prescription at the end of the data collection period. Data on residents and UTI episodes were provided anonymously. Each physician reviewed cases separately based on clinical criteria for UTI, prescription guidelines provided (supplement Table 2, supplement 3) and on published criteria for initiating antibiotics. Discrepancies were resolved in discussion.

### Outcomes

The primary outcome was the proportion of adequate prescriptions (adequate in terms of antimicrobial choice and dosage; termed “adequate antibiotic choice”).

Secondary outcomes were:

- proportion of adequate prescriptions (adequate in terms of decision to treat; termed “adequate indication for treatment”).
- proportion of quinolones used for UTI without indwelling urinary catheter.
- proportion of urinary cultures performed.
- proportion of cases with clinical failure (defined as need for additional antimicrobial treatment for UTI within 7 days of previous episode).
- proportion of admissions to hospital due to UTI.
- proportion of adverse events attributed to antimicrobial treatment for UTI.

The last three outcomes were intended as safety outcomes.

The proportion of nursing and medical staff who underwent training was recorded.

### Statistical analysis

To investigate the primary hypothesis whether the frequency of adequate prescriptions are different in both groups generalized linear models (probability distribution: binomial; link function: log) were used to estimate adjusted risk ratios (RR) with 95%CI. The unit of analysis was the patient, which were nested within centres. Two reviewers evaluated the adequacy of prescription. Inter-reviewer consistency was evaluated comparing the evaluation of both reviewers. Therefore Cohen’s ĸ and 95% confidence intervals of Cohen’s ĸ were calculated.

Three study periods (pre-intervention period, intervention-period, post-intervention period) were analysed separately.

Secondary outcomes (adequate indication for treatment, quinolone use for UTI without catheter, urinary culture performed, clinical failure, hospital admission due to UTI) were analysed in the same way. Similar to the primary outcome adequate decision to treat was evaluated by two reviewers.

Data including demographic and baseline characteristics were compared using χ²-test or Fisher’s-exact-test for categorical variables and t-test or Mann-Whitney-U-test for continuous variables depending on if the data were normally distributed or skewed. A P-value < 0.05 was considered statistically significant. Statistical analyses were performed using SAS 9.4 (2002–2012 by SAS Institute Inc., Cary, NC, USA).

## Results

The characteristics of LTCFs of both groups are summarized in Table 1.

During the study, 326 UTI episodes were recorded, 161 in the intervention group and 165 in the control group. In the pre-intervention phase, we recorded 71 UTI episodes. During the intervention and post-intervention period, 167 and 88 episodes were documented, respectively. In the control group, 55/165 (33%) of UTIs were recorded in patients with an indwelling urinary catheter compared to 22/161 (14%) in the intervention group (*p* < 0.004). There was a statistically significant difference in the method of communication between LTCF and physicians. In the intervention group, the physician was present at the LTCF in 26/161 (16%) of cases of UTI compared to 72/165 (44%) in the control group. In contrast, physicians were contacted by phone or fax 135/165 (84%) in the intervention group and in 93/165 (56%) in the control group (*p* < 0.001).

### Patient characteristics

UTIs were diagnosed in 194 patients. There were no significant differences between the study populations, i.e. patients with UTIs, in terms of age, sex, history of allergies to antimicrobial substances, history of renal impairment or urologic diseases (Table 2). There was a statistically significant difference in reported weight (control group median 66 kg (range 38–135 kg) vs. intervention group median 59 kg (range 38–143 kg), *p* = 0.014).

### Intervention reach

During the intervention period (April 12, 2021– November 3, 2021), 30 onsite trainings were conducted by ICP team. Overall, 209 nursing staff members took part in these trainings. On October 31, 2021, the number of nursing staff employed by the four LTCFs of the intervention group was 205. The discrepancy between team members who took part in the trainings and the number of employees as of October 31, 2021 can be explained by staff turnover. All physicians received written information about the project during their visits at the LTCFs including the guideline and information on the website at five time points throughout the study. They were invited to the online educational sessions on the UTI program. Half of the physicians (15/ 30) attended at least one of the online sessions.

### Primary outcome

Inter-reviewer consistency was high for the primary outcome (ĸ = 0.98; 95%CI 0.96-1.00). Out of 326 UTI episodes 323 (99.1%) were evaluated the same. Only in three UTI episodes discordant evaluations were observed. Due to the high level of agreement between the two reviewers, the two evaluations were combined for analysis. Therefore, adequate prescription was defined as follows: both reviewers evaluated the prescription adequate.

**Fig. 1:**
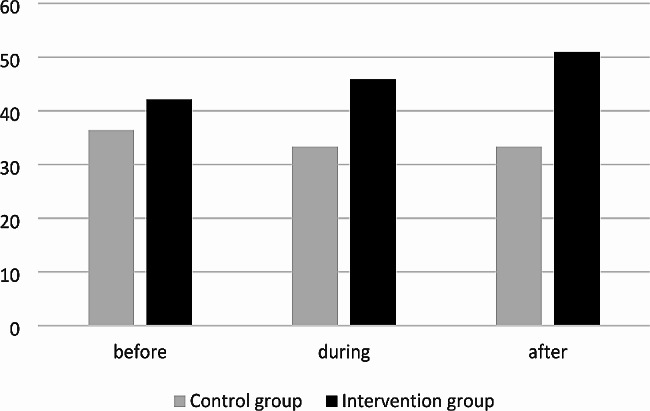
Proportion of adequate antimicrobial treatments (adequate in terms of choice) before, during and after interventions

In the intervention group, the proportion of adequate antibiotic choices increased from 42.1% in the pre-intervention period, to 45.9% during the intervention and to 51% in the post-intervention period (absolute increase of 8.9%). In the control group, the proportion was 36.4%, 33.3% and 33.3%, respectively (Fig. 1). Therefore, the numerical difference between intervention group and control group in the post-intervention period was 17.7%. However, the differences between intervention group and control group did not reach statistical significance (Tables 3 and 4).

**Table 4:** Summary of results (raw numbers and proportions)

Study period	Before intervention	During intervention	After intervention	
Groups	Intervention vs. control	Intervention vs. control	Intervention vs. control	
Primary outcome				
Adequate choice of antimicrobial	16/38 (42.1%) vs. 12/33 (36.4%)	34/74 (45.9%) vs. 31/93 (33.3%)	25/49 (51%) vs. 13/39 (33.3%)	
Secondary outcomes				
Adequate decision to treat	27/38 (71.1%) vs. 25/33 (75.8%)	66/74 (89.2%) vs. 67/93 (72%)	44/49 (89.8%) vs. 35/39 (89.7%)	
Quinolone use for UTI without catheter	2/32 (6.3%) vs. 6/19 (31.6%)	6/59 (10.2%) vs. 8/49 (16.3%)	4/42 (9.5%) vs. 7/26 (26.9%)	
Urinary culture performed	7/38 (18%) vs. 1/33 (3%)	11/74 (14.9%) vs. 2/92 (2.2%)	7/49 (14.3%) vs. 0/39	
Clinical failure	5/40 (12.5%) vs. 3/34 (8.8%)	9/71 (12.7%) vs. 11/92 (12%)	3/48 (6.3%) vs. 0/48	
Hospital admission due to UTI	3/38 (7.9%) vs. 3/33 (9.1%)	5/74 (6.8%) vs. 4/93 (4.3%)	8/49 (16.3%) vs. 1/39 (2.6%)	

### Secondary outcomes

**Fig. 2:**
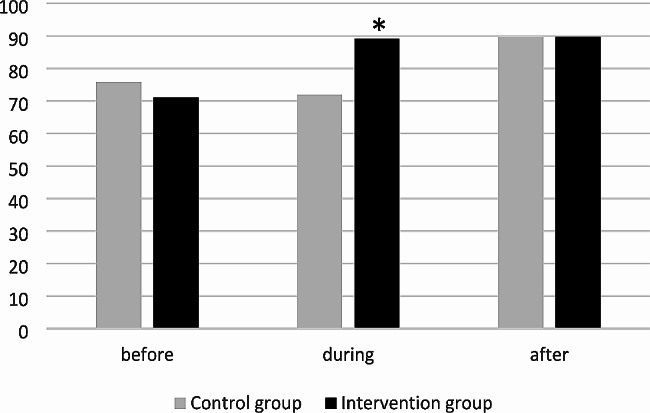
Proportion of adequate antimicrobial treatments (adequate decision to treat) before, during and after the interventions. *p < 0.05 comparing intervention and control group during the intervention period

Because agreement in secondary outcomes was also high (ĸ = 0.97; 95%CI 0.93-1.00; concordant evaluations: 99.1%) the two evaluations were combined for analysis in the same way as for the primary outcome. During the intervention period, the risk ratio (RR) for inadequate indication for treatment was 0.41 (95% CI 0.19–0.90), *p* = 0.025 (Fig. 2; Table 3). In the post-intervention period, there was no difference between intervention group and control group, due to an increase in adequate indications for treatment in the control group (from 72.0% in the intervention period to 89.7% in the post-intervention period).

During the study, 227 (69.6%) UTI episodes without an indwelling urinary catheter were recorded, 133 in the intervention group and 94 in the control group. Before and after the intervention period, the risk ratio (RR) for use of quinolones for UTI without indwelling urinary catheters were significantly reduced (RR: 0.17, 95%CI: 0.04–0.72; and 0.18; 95%CI: 0.04–0.89, respectively. Tables 3 and 4).

During the study period, 28 (8.6%) urinary cultures were performed (326 UTI episodes recorded). Throughout the study, more urinary cultures were ordered in the IG compared to control group. This difference was statistically significant during the intervention period (RR 6.89, 95%CI 1.59–29.83, *p* = 0.010, Table 3). In the post-intervention period, a statistical analysis was not possible as no cultures were performed in the control group (compared to 7 in the intervention group).

Our safety outcomes were: proportion of clinical failure (defined as need for additional antimicrobial treatment for UTI within 7 days of previous episode), number of hospital admissions due to UTI and adverse events due to antimicrobial treatment. There were no significant differences between the control group and intervention group in the safety outcomes (Tables 3 and 4). Only one adverse event due to antimicrobial treatment was documented during the entire study period (in the intervention group in the pre-intervention period).

## Discussion

In this non-randomized cluster-controlled intervention study in LTCFs in Austria, a multifaceted bundle of antimicrobial stewardship interventions consisting of clinical practice guidelines, local as well as web-based education targeting nursing staff and primary care physicians resulted in a significant increase in adequate indications for treatment during the intervention period. We found a higher number of adequate antibiotic choices for UTIs with a numerical difference between the intervention group and control group of 17.7% in the post-intervention phase. However, this difference did not reach statistical significance. The interventions did not lead to an increase in proportion of clinical failures, hospital admissions due to UTI or adverse events due to antimicrobial treatment.

During the intervention period, the risk ratio for inadequate treatment (in terms of decision to treat the UTI) was 0.41 (*p* = 0.025), indicating that the interventions led to a change in prescription culture. The proportion of adequate indications for treatment remained high (89%) in the intervention group in the post-intervention phase, suggesting an effect also during this phase. However, there was no significant difference between intervention group and control group in the post-intervention period, due to an increase in adequate indications for treatment in the control group (from 72.0% in the intervention period to 89.7% in the post-intervention period). The reasons for this increase in the control group remain unclear. Although the intervention was restricted to LTCFs in Graz and controls were located outside of the city, we cannot completely rule out a partial spill over of the intervention within the trans-regional physician community.

Quinolone use for treatment of UTIs without indwelling urinary catheter was significantly lower in the intervention group. However, this difference existed already before our interventions and persisted thereafter. This difference could reflect a different prescription culture between physicians serving at LTCFs in the intervention group and control group. In 2018, systematic surveillance of health-care associated infections was initiated in the LTCFs of the Geriatric Health Centers Graz (intervention group ) [16]. Antimicrobial prescriptions were also recorded. Implementation of this surveillance system could have contributed to physicians’ awareness on negative aspects of quinolone use in elderly patients. Several other aspects shed light on differences in daily practice between the LTCFs in the two groups. In the control group, significantly more UTIs were recorded in patients with an indwelling urinary catheter (33% compared to 14% in the IG, *p* < 0.004). This could indicate a higher use of urinary catheters in the control group overall. In addition, physicians prescribing antibiotics for UTIs were physically present in 16% of cases in intervention group compared to 44% in the control group. In the intervention group, physicians were contacted mainly by phone or fax. Our findings are in contrast to a study from Northern Ireland reporting that between 58% and 70% of systemic antimicrobial prescriptions were initiated following a physician’s visits at the LTCF [17]. The fact that physicians were mainly contacted by phone or fax before prescribing antibiotics for a UTI underlines the importance of the role of nursing staff in the prescription process as has been described before [18]. Knowledge on UTIs and communication skills of nurses as well as stable staffing and continuity of care play an important role in adequate reporting of signs and symptoms suggestive of UTIs in residents [19].

Even though urinary cultures are recommended as part of the diagnostic procedures for complicated UTIs [20], only 3 urinary cultures were ordered during the entire study period in the control group. Logistic considerations such as limited access to microbiological tests and the length of time needed to obtain culture results could explain this finding in the control group as has been described previously [18]. Our finding of very low utilization of urinary cultures is contrasted by a study by Brown et al. which successfully aimed at reducing unnecessary urinary cultures in Canadian LTCFs starting from a baseline of 3.2 urinary cultures per 1000 resident days [21].

The reach of our interventions among nursing staff was high, as every member of nursing staff attended one onsite training. Every physician received written information on the project several times during the study period during their visits at the LTCFs. This included the guideline that was written in co-operation with a general practitioner active at one of the LTCFs. Unfortunately, only half of the physicians attended at least one of the online sessions. The differences in reach of the intervention between nursing staff and physicians can probably be explained by the fact that nursing staff is employed by the LTCFs whereas the general practitioners are not. In Austria, general practitioners typically work as freelancers. The outcomes could probably have improved if the reach within the physician community had been better.

Several studies describe successful antibiotic stewardship interventions for the prescription of antimicrobial substances for UTIs in long-term care facilities in different settings and countries [22–27]. Different intervention strategies were used: audit and feedback [22], guidelines and implementation of interdisciplinary quality circles [23], educational sessions [24] and implementation of local ASB teams [26]. The primary outcomes were mainly number of antimicrobial prescriptions for UTI/ time period [24, 25] or overall use of antimicrobials [23, 26]. Our study focused a qualitative improvement of antimicrobial prescriptions for UTIs rather than an overall reduction of actual prescriptions. Therefore, a direct comparison of results is difficult. We selected our outcomes based on a previous study indicating a relatively low number of health-care associated infections in our intervention group compared to data from the literature [16]. Therefore, it appeared less promising to attempt a decrease in the number of antimicrobial prescriptions compared to improving the quality of prescriptions.

Our study has several limitations. We used a non-randomized cluster-controlled study design because randomisation of LTCFs was not feasible as several general practitioners cared for patients in more than one LTCF in Graz. We therefore chose LTCFs in another region to avoid spillover of the intervention. Voluntary participation of LTCFs might mean generalisability of our findings is limited to LTCFs interested in antibiotic stewardship. Our study was conducted during the COVID-19 pandemic. As this was a demanding period for nursing staff and general practitioners (e.g. handling COVID-19 cases at the LTCFs, shortage of staff due to illness, etc.) the impact of the interventions may have been reduced.

## Conclusion

Antimicrobial stewardship interventions consisting of clinical practice guidelines, local as well as web-based education for nursing staff and primary care physicians resulted in a significant increase in adequate indications for treatment ) during the intervention period. However, this difference was not maintained in the post-intervention phase. The interventions did not lead to an increase in proportion of clinical failures, hospital admissions due to UTI or adverse events due to antimicrobial treatment. Continued efforts to improve the quality of prescriptions further are necessary.

### Electronic supplementary material

Below is the link to the electronic supplementary material.

Supplementary Material 1

## Data Availability

The datasets analysed during the current study are available from the corresponding author on reasonable request.

## Abbreviations

LTCFs: long-term care facilities
UTIs: urinary tract infections
IG: intervention group
CG: control group
IRB: institutional review board
RR: risk ratio

## References

[1] Costelloe C, Metcalfe C, Lovering A, Mant D, Hay AD (2010). Effect of antibiotic prescribing in primary care on antimicrobial resistance in individual patients: systematic review and meta-analysis. BMJ.

[2] Goossens H, Ferech M, Vander Stichele R, Elseviers M, Group EP (2005). Outpatient antibiotic use in Europe and association with resistance: a cross-national database study. Lancet.

[3] Antimicrobial Resistance. Tackling a crisis for the health and wealth of nations. 2014.

[4] Ribbe MW, Ljunggren G, Steel K, Topinkova E, Hawes C, Ikegami N (1997). Nursing homes in 10 nations: a comparison between countries and settings. Age Ageing.

[5] Strausbaugh LJ (2001). Emerging health care-associated infections in the geriatric population. Emerg Infect Dis.

[6] Hepper HJ, Sieber C, Walger P, Bahrmann P, Singler K (2013). Infections in the elderly. Crit Care Clin.

[7] Moro ML, Jans B, Cookson B, Fabry J (2010). The burden of healthcare-associated infections in European long-term care facilities. Infect Control Hosp Epidemiol.

[8] Mody L, Crnich C (2015). Effects of Excessive Antibiotic use in nursing homes. JAMA Intern Med.

[9] Nicolle LE, Bentley DW, Garibaldi R, Neuhaus EG, Smith PW (2000). Antimicrobial use in long-term-care facilities. SHEA Long-Term-Care Committee. Infect Control Hosp Epidemiol.

[10] Ricchizzi E, Latour K, Karki T, Buttazzi R, Jans B, Moro ML et al. Antimicrobial use in European long-term care facilities: results from the third point prevalence survey of healthcare-associated infections and antimicrobial use, 2016 to 2017. Euro Surveill. 2018;23(46). 10.2807/1560-7917.ES.2018.23.46.1800394 PMC6247460 30458913

[11] Benoit SR, Nsa W, Richards CL, Bratzler DW, Shefer AM, Steele LM (2008). Factors associated with antimicrobial use in nursing homes: a multilevel model. J Am Geriatr Soc.

[12] Loeb M, Simor AE, Landry L, Walter S, McArthur M, Duffy J (2001). Antibiotic use in Ontario facilities that provide chronic care. J Gen Intern Med.

[13] Rotjanapan P, Dosa D, Thomas KS (2011). Potentially inappropriate treatment of urinary tract infections in two Rhode Island nursing homes. Arch Intern Med.

[14] Peron EP, Hirsch AA, Jury LA, Jump RL, Donskey CJ (2013). Another setting for stewardship: high rate of unnecessary antimicrobial use in a veterans affairs long-term care facility. J Am Geriatr Soc.

[15] Moro ML, Gagliotti C (2013). Antimicrobial resistance and stewardship in long-term care settings. Future Microbiol.

[16] Konig E, Medwed M, Pux C, Uhlmann M, Schippinger W, Krause R et al. Prospective Surveillance of Healthcare-Associated Infections in residents in Four Long-Term Care facilities in Graz, Austria. Antibiot (Basel). 2021;10(5). 10.3390/antibiotics10050544 PMC8151996 34067175

[17] McClean P, Tunney M, Gilpin D, Parsons C, Hughes C (2012). Antimicrobial prescribing in residential homes. J Antimicrob Chemother.

[18] van Buul LW, van der Steen JT, Doncker SM, Achterberg WP, Schellevis FG, Veenhuizen RB (2014). Factors influencing antibiotic prescribing in long-term care facilities: a qualitative in-depth study. BMC Geriatr.

[19] Kirsebom M, Hedstrom M, Poder U, Wadensten B (2017). General practitioners’ experiences as nursing home medical consultants. Scand J Caring Sci.

[20] Bonkat G, EAU Guidelines on Urological Infections.: European Association of Urology 2023 https://d56bochluxqnz.cloudfront.net/documents/full-guideline/EAU-Guidelines-on-Urological-infections-2023.pdf.

[21] Brown KA, Chambers A, MacFarlane S, Langford B, Leung V, Quirk J (2019). Reducing unnecessary urine culturing and antibiotic overprescribing in long-term care: a before-and-after analysis. CMAJ Open.

[22] Doernberg SB, Dudas V, Trivedi KK (2015). Implementation of an antimicrobial stewardship program targeting residents with urinary tract infections in three community long-term care facilities: a quasi-experimental study using time-series analysis. Antimicrob Resist Infect Control.

[23] Pluss-Suard C, Niquille A, Hequet D, Krahenbuhl S, Pichon R, Zanetti G (2020). Decrease in Antibacterial Use and Facility-Level Variability after the introduction of guidelines and implementation of physician-pharmacist-nurse quality circles in Swiss Long-Term Care facilities. J Am Med Dir Assoc.

[24] Arnold SH, Nygaard Jensen J, Bjerrum L, Siersma V, Winther Bang C, Brostrom Kousgaard M (2021). Effectiveness of a tailored intervention to reduce antibiotics for urinary tract infections in nursing home residents: a cluster, randomised controlled trial. Lancet Infect Dis.

[25] Hartman EAR, van de Pol AC, Heltveit-Olsen SR, Lindbaek M, Hoye S, Lithen SS (2023). Effect of a multifaceted antibiotic stewardship intervention to improve antibiotic prescribing for suspected urinary tract infections in frail older adults (ImpresU): pragmatic cluster randomised controlled trial in four European countries. BMJ.

[26] Penalva G, Crespo-Rivas JC, Guisado-Gil AB, Rodriguez-Villodres A, Pachon-Ibanez ME, Cachero-Alba B (2023). Clinical and ecological impact of an Educational Program to optimize antibiotic treatments in nursing homes (PROA-SENIOR): a cluster, Randomized, controlled trial and interrupted time-series analysis. Clin Infect Dis.

[27] Pettersson E, Vernby A, Molstad S, Lundborg CS (2011). Can a multifaceted educational intervention targeting both nurses and physicians change the prescribing of antibiotics to nursing home residents? A cluster randomized controlled trial. J Antimicrob Chemother.

