# Supplementary material for: Implementation of an antimicrobial stewardship program for urinary tract infections in long-term care facilities: a cluster-controlled intervention study
Source: Antimicrob Resist Infect Control. 2024 Apr 16;13:43. doi: 10.1186/s13756-024-01397-2 (PMC11020885; doi:10.1186/s13756-024-01397-2)
Supplement: Supplementary file 1 — Supplementary Material 1 [file 13756_2024_1397_MOESM1_ESM.docx]

**Supplements**

Supplement table 1 Criteria to obtain urine specimen (adapted from (22))

| Resident without indwelling catheter |
| --- |
| Presence of UTI symptoms in residents with predisposing factors for complicated UTI (e.g. after urological surgery, nephrolithiasis, etc.) |
| Recurrent UTIs (≥2 episodes in 6 months, ≥3 episodes in 12 months) |
| Persisting symptoms after or during antimicrobial therapy |
| Unexplained febrile illness |
| Resident with indwelling catheter |
| Presence of UTI symptoms |
| Recurrent UTIs (≥2 episodes in 6 months, ≥3 episodes in 12 months) |
| Persisting symptoms after or during antimicrobial therapy |
| Unexplained febrile illness |

Supplement table 2 Clinical criteria for UTI (based on (23))

| Resident without indwelling catheter |
| --- |
| Acute dysuria |
| New or marked increase in frequency or urgency |
| New or marked increase in incontinence |
| Acute change in metal status from baseline |
| Gross hematuria |
| Fever (> 1.1°C from baseline) or chills (without signs of other infection) |
| Resident with indwelling catheter |
| Fever (> 1.1°C from baseline), chills, hypotension (without signs of other infection) |
| Acute change in metal status or functional decline from baseline |
| New-onset suprapubic or costovertebral angle pain |
| Purulent discharge from around the catheter or acute pain or swelling of testes, epididymis or prostate |

Supplement 3a: Guidelines as distributed for UTIs with and without indwelling catheters

**Diagnostics and antibiotic therapy for urinary tract infections (UTI)**

**WITHOUT an indwelling urinary catheter**

**Possible clinical symptoms of UTI in geriatric patients^1,2^:**

- Burning/painful urination, lower abdominal pain
- Newly increased frequency of urination
- New or increasing incontinence
- Fever (without signs of another infection)
- Nausea, vomiting
- Behavioral abnormalities/delirium
- Vigilance disorders/fatigue/weakness
- Blood-stained urine

**Indications for urine culture^3^:**

- Signs of UTI in the presence of predisposing factors (e.g. urinary obstructions)
- Signs of recurrent UTI (≥2 episodes in 6 months, ≥3 episodes in 12 months)
- Persistence of symptoms during or after antibiotic therapy
- Fever of unknown origin

**Empirical therapy: acute uncomplicated cystitis** ^2,3,4^

First choice: Fosfomycin/Trometamol (e.g. Monuril) 1x3g, day 1 (possibly additionally day 3 and 5)

Nitrofurantoin (e.g. Furadatin ret) 2x 100mg (contraindicated in chronic kidney disease*)

Pivmecillinam (e.g. Selexid) 2-3 x 400mg

Alternatives**: Amoxicillin/clavulanic acid (z.B. Augmentin, Xiclav) 2-3x 1000mg
Cefalexin (z.B. Ospexin) 2-3 x 1000mg
Ciprofloxacin (z.B. Ciproxin) 2x 250mg
Levofloxacin (z.B. Tavanic) 1x 500mg
Trimethoprim (z.B. Motrim) 2x200mg

Duration of therapy: 3-5 days

* glomerular filtration rate < 45 ml/min

** Fluoroquinolones and Cephalosporins have the highest risk of *Clostridium difficile* infection and selection of multidrug-resistant pathogens.^4^

If you have any questions regarding culture results, treatment options, etc., please contact:

Geriatric Consultation Service or Section for Infectiology, Medical University of Graz

References:

1. https://ecdc.europa.eu/sites/portal/files/media/en/publications/Publications/HALT-3-LTCF-PPS-Protocol-v2.1.pdf
2. Leitlinie zur Behandlung von HWI und Atemwegsinfektionen, 2017, Geriatrische Gesundheitszentren der Stadt Graz
3. http://www.oeginfekt.at/download/cs-akuter_hwi.pdf
4. S3 Leitlinie 2017, <https://www.awmf.org/uploads/tx_szleitlinien/043-044k_S3_Harnwegsinfektionen_2017-05.pdf>

Created on: 04.10.2019 by: Ines Zollner-Schwetz, Elisabeth Ullrich, Eric Stoiser, Christian Pux, Michael Wendler

**Diagnostics and antibiotic therapy for urinary tract infections (UTI)**

**WITH an indwelling urinary catheter**

**Possible clinical symptoms of UTI in geriatric patients ^,2^:**

- Fever, chills OR new onset of hypotension WITHOUT another infection
- Acute change in level of consciousness OR acute functional deterioration WITHOUT other diagnosis
- New onset of suprapubic pain or pain in the kidney area (pressure sensitive) or in the lower abdomen or flank palpitations
- Purulent discharge around the catheter or acute pain, swelling or tenderness of the testicles, epididymis or prostate
- New onset of hematuria

Presence or absence of foul odor or urinary turbidity does not allow differentiation between UTI and asymptomatic bacteriuria ^2^.

**Indications for urine culture^3^:**

- Signs of a urinary tract infection (including first occurrence)
- Signs of recurrent UTI (≥2 episodes in 6 months, ≥3 in 12 months)
- Persistence of symptoms during or after antibiotic therapy
- Fever of unknown origin.

Routine urine cultures in asymptomatic patients are NOT recommended.

**Procedure for suspected urinary tract infection with an indwelling urinary catheter:**

- Collection of urine for a urine culture BEFORE initiating antibiotic therapy ^2,4^.
- Always change the urinary catheter, especially in cases where it has been in place for longer than 7 days, obtain urine from newly placed catheter for culture ^4 ,5^.
- If urinary catheter cannot be changed on site, aseptic collection of urine from the collection tube of the urinary catheter.
- Initiate empirical therapy with Ciprofloxacin (e.g. Ciproxin) 2x 500mg po or Amoxicillin/Clavulanic acid (e.g. Augmentin, Xiclav) 2-3x 1000mg po (depending on previous findings).
- Adapt the antibiotic therapy to the culture result.
- In case of hypotension or significant deterioration in general condition, consider hospitalization for intravenous therapy.

Duration of therapy: 7 days in good treatment response, up to 14 days with a delayed response^2^

If you have any questions regarding culture results, treatment options, etc., please contact:

Geriatric Consultation Service or Section for Infectiology, Medical University of Graz

References:

1. https://ecdc.europa.eu/sites/portal/files/media/en/publications/Publications/HALT-3-LTCF-PPS-Protocol-v2.1.pdf
2. Guideline on Urological Infections, European Association of Urology https://uroweb.org/guideline/urological-infections/#3
3. http://www.oeginfekt.at/download/cs-akuter_hwi.pdf
4. https://www.nice.org.uk/guidance/ng113
5. European and Asian guidelines on management and prevention of catheter-associated urinary tract infections, Tenke P, et al. , International Journal of Antimicrobial Agents, 2008

Created on: 4.10. 2019by: Ines Zollner-Schwetz, Elisabeth Ullrich, Eric Stoiser, Christian Pux, Michael Wendler

Supplement 3b: Guidelines as distributed for UTIs with and withoud indewlling catheters in German

**Diagnostik und Antibiotika-Therapie bei Harnwegsinfektionen**

**OHNE liegenden Harndauerkatheter**

**Mögliche klinische Symptome eines HWI beim geriatrischen Patienten bzw. Patientin^1,2^:**

- Brennen/ Schmerzen beim Urinieren, Unterbauchschmerzen
- Neu aufgetretene erhöhte Frequenz beim Wasserlassen
- Neu aufgetretene oder zunehmende Inkontinenz
- Fieber (ohne Anzeichen für eine andere Infektion)
- Übelkeit, Erbrechen
- Verhaltensauffälligkeiten/Delir
- Vigilanzstörungen/Müdigkeit/Mattigkeit
- blutiger Harn

**Indikationen für eine Harnkultur^3^:**

- Anzeichen eines HWI bei disponierenden Faktoren (z.B. Abflusshindernisse)
- Anzeichen eines rezidivierenden HWI (≥2 Episoden in 6 Monaten, ≥3 Episoden in 12 Monaten)
- Fortbestehen der Symptome unter bzw. nach Antibiotikatherapie
- Fieber unklarer Genese

**Empirische Therapie: akute unkomplizierte Zystitis** ^2,3,4^

Erste Wahl: Fosfomycin/Trometamol (z.B. Monuril) 1x3g, Tag 1 (ggf. zusätzlich Tag 3 und 5) Nitrofurantoin (z.B. Furadatin ret) 2x 100mg (kontraindiziert bei Niereninsuffizienz*) Pivmecillinam (z.B. Selexid) 2-3 x 400mg

Alternativen**: Amoxicillin/Clavulansäure (z.B. Augmentin, Xiclav) 2-3x 1000mg
Cefalexin (z.B. Ospexin) 2-3 x 1000mg
Ciprofloxacin (z.B. Ciproxin) 2x 250mg
Levofloxacin (z.B. Tavanic) 1x 500mg
Trimethoprim (z.B. Motrim) 2x200mg

Therapiedauer: 3-5 Tage
* ab glomerulärer Filtrationsrate < 45 ml/min
**Bei Fluorchinolonen und Cephalosporinen ist das Risiko für eine Clostridium difficile Infektion und für die Selektion von multiresistenten Erregern am höchsten.^4^

Referenzen:

1. https://ecdc.europa.eu/sites/portal/files/media/en/publications/Publications/HALT-3-LTCF-PPS-Protocol-v2.1.pdf
2. Leitlinie zur Behandlung von HWI und Atemwegsinfektionen, 2017, Geriatrische Gesundheitszentren der Stadt Graz
3. http://www.oeginfekt.at/download/cs-akuter_hwi.pdf
4. S3 Leitlinie 2017, https://www.awmf.org/uploads/tx_szleitlinien/043-044k_S3_Harnwegsinfektionen_2017-05.pdf

Erstellt am: 04.10.2019

von: Ines Zollner-Schwetz, Elisabeth Ullrich, Eric Stoiser, Christian Pux, Michael Wendler

**Diagnostik und Antibiotika-Therapie bei Harnwegsinfektionen**

**MIT liegendem Harndauerkatheter**

**Mögliche klinische Symptome eines HWI beim geriatrischen Patienten bzw. Patientin^1,2^:**

- Fieber, Schüttelfrost ODER neu aufgetretene Hypotonie OHNE eine andere Infektion
- Akute Veränderung der Bewusstseinslage ODER akute funktionelle Verschlechterung OHNE andere Diagnose
- Neu aufgetretener suprapubischer Schmerz oder Schmerzen im Nierenlager oder Druckschmerz im Unterbauch oder Flankenklopfschmerz
- Eitriger Ausfluss im Bereich des Katheters oder akuter Schmerz, Schwellung oder Druckschmerz der Hoden, Nebenhoden oder Prostata
- Neu aufgetretene Hämaturie.

Vorhandensein oder Fehlen von üblem Geruch oder Harntrübung erlaubt keine Unterscheidung zwischen HWI und asymptomatischer Bakteriurie ^2^.

**Indikationen für eine Harnkultur^3^:**

- Anzeichen eines Harnwegsinfekts (auch bei erstmaligem Auftreten)
- Anzeichen eines rezidivierenden HWI (≥2 Episoden in 6 Monaten, ≥3 in 12 Monaten)
- Fortbestehen der Symptome unter bzw. nach Antibiotikatherapie
- Fieber unklarer Genese.

Routineharnkulturen bei asymptomatischen Patienten werden NICHT empfohlen.

**Vorgehen bei Verdacht auf Harnwegsinfekt bei liegendem Harndauerkatheter:**

- Abnahme von Harn für eine Harnkultur VOR Einleitung der Antibiotikatherapie ^2,4^.
- Harnkatheter immer wechseln, auf jeden Fall wenn länger als 7 Tage liegend, Harn aus dem frischen Katheter für Kultur verschicken ^4 ,5^.
- Wenn Harnkatheter vor Ort nicht gewechselt werden kann, aseptische Abnahme von Harn aus dem Entnahmeschenkel des Harnkatheters.
- Einleiten einer empirischen Therapie mit Ciprofloxacin (z.B. Ciproxin) 2x 500mg po oder Amoxicillin/Clavulansäure (z.B. Augmentin, Xiclav) 2-3x 1000mg po (abh. von Vorbefunden)
- Anpassen der Antibiotikatherapie an das Kulturergebnis.
- Bei Hypotonie oder deutlicher Verschlechterung des Allgemeinzustandes Krankenhauseinweisung zur i.v. Therapie erwägen.

Therapiedauer: 7 Tage bei gutem Ansprechen, bis 14 Tage bei verzögertem Ansprechen ^2^

Referenzen:

1. https://ecdc.europa.eu/sites/portal/files/media/en/publications/Publications/HALT-3-LTCF-PPS-Protocol-v2.1.pdf
2. Guideline on Urological Infections, European Association of Urology https://uroweb.org/guideline/urological-infections/#3
3. http://www.oeginfekt.at/download/cs-akuter_hwi.pdf
4. https://www.nice.org.uk/guidance/ng113
5. European and Asian guidelines on management and prevention of catheter-associated urinary tract infections, Tenke P, et al. , International Journal of Antimicrobial Agents, 2008

Erstellt am 4.10. 2019
von: Ines Zollner-Schwetz, Elisabeth Ullrich, Eric Stoiser, Christian Pux, Michael Wendler

Supplement table 4: Study periods

|  | Control | Intervention |
| --- | --- | --- |
| Pre-intervention | March 1, 2021 – June 11, 2021 | January 1, 2021 –April 11, 2021 |
| Intervention | June 12, 2021 – January 3, 2022 | April 12, 2021 – November 3, 2021 |
| Post-intervention | January 4, 2022 – 24.June 2022 | November 4, 2021-April 24, 2022 |
